# 4D (space + time) datasets of spruce wood enzymatic hydrolysis

**DOI:** 10.1016/j.dib.2026.112489

**Published:** 2026-01-20

**Authors:** Solmaz Hossein Khani, Maxime Corré, Khadidja Ould Amer, Noah Remy, Berangère Lebas, Anouck Habrant, Gabriel Paës, Yassin Refahi

**Affiliations:** Université de Reims-Champagne-Ardenne, INRAE, FARE, UMR A 614, Reims, France

**Keywords:** Enzymatic deconstruction, Autofluorescence, Image processing, Drift compensation, Segmentation

## Abstract

The conversion of lignocellulosic biomass from plant cell walls into bioproducts can contribute to reducing dependence on fossil sources and achieving sustainable development. Biotechnological conversion of lignocellulosic biomass has several advantages over other conversion approaches such as thermochemical and chemical conversions. These advantages include improved efficiency and specificity for desired products, ecological compatibility and reduced toxicity. Enzymatic transformation is a key step in biotechnological conversion. To achieve a cost-effective conversion, a comprehensive understanding of cell wall enzymatic hydrolysis is required. Despite progress, the enzymatic hydrolysis at microscale is comparatively understudied and lacks comprehensive investigation. Addressing this gap requires collection of time-lapse image datasets of cell wall enzymatic hydrolysis which is a technically demanding task. Furthermore, accurate processing of the time-lapse images to identify and track individual cell walls is particularly challenging, notably because of the sample drift present in the images. Recently, an efficient image processing pipeline, called AIMTrack, has been developed which uses an enhanced divide-and-conquer strategy to divide time-lapse images into clusters whose sizes are dynamically adjusted to the deconstruction extent. The image registrations are then limited to clusters and the resulting transformations are combined to correct sample drift across time-lapse images. Subsequently AIMTrack provides segmentation of time-lapse images where voxels belonging to the same cell walls are labelled with a unique identifier. The time-lapse image datasets presented here consist of time-lapse images of spruce wood cell walls acquired during enzymatic hydrolysis using a cellulolytic enzyme cocktail at two enzyme loadings of 15 and 30 FPU/g biomass. Control time-lapse datasets which are acquired under the identical conditions, but without addition of enzymes, are also included. Both control and hydrolysis datasets are processed using AIMTrack to track the cell walls from time-lapse images. The generated segmentations are also provided.

Specifications Table

**Table:** 

Subject	Biotechnology
Specific subject area	Plant biotechnology, Confocal fluorescence imaging, Enzymatic deconstruction, Recalcitrance, Image processing
Type of data	Image Raw and Processed
Data collection	Pretreated spruce wood *(Picea abies)* sections incubated with a diluted cellulolytic enzymatic cocktail were imaged during enzymatic deconstruction for 24 h using a fluorescence confocal microscope. Control datasets were also collected in the same conditions without the enzymatic cocktail. The acquired time-lapse images were analysed using AIMTrack, an image processing pipeline to analyse time-lapse images of highly deconstructed samples. AIMTrack incorporates an efficient drift compensation method to ensure accuracy. AIMTrack generated the cell wall segmentations of the acquired time-lapse image datasets.
Data source location	The time-lapse image datasets were acquired and processed at FARE laboratory, Reims, France.
Data accessibility	Repository name: Zenodo Data identification number: 10.5281/zenodo.17234278 Direct URL to data: https://zenodo.org/records/17234278 Instructions for accessing these data: The time-lapse acquisitions and associated segmentations are available as a single downloadable .zip file using the above link; the folder and file structure is detailed in the *Data Description* section.
Related research article	S. Hossein Khani, K. Ould Amer, F. Shiasi Ghalemaleki, M. Corré, N. Remy, A. Habrant, B. Lebas, J. Massah, A. Faraj, G. Paës, & Y. Refahi. “Plant cell wall enzymatic hydrolysis: Predicting yield dynamics from autofluorescence and morphological temporal changes”. *Bioresour. Technol.*, 442 (2026), 133642. 10.1016/j.biortech.2025.133642

## 1 Value of the Data

• The presented time-lapse images provide microscale datasets of plant cell wall enzymatic deconstruction which is relatively underexamined at microscale. The datasets can contribute to achieve a comprehensive understanding of cell wall enzymatic hydrolysis which is required for an economically viable conversion of lignocellulosic biomass.
• The collection of time-lapse images of cell wall enzymatic hydrolysis is both time-consuming and technically demanding. The presented datasets include 24-hour time-lapse of samples subjected to enzymatic deconstruction at two enzyme loadings of 15 and 30 FPU/g biomass together with control datasets without enzymes.
• The datasets include segmented time-lapse images for tracking individual cell walls during hydrolysis, with expert validation and correction. Accurate segmentation of 4D image datasets is challenging, notably because of sample drift and deformation.
• The datasets can be analysed to investigate cell wall autofluorescence dynamics and morphodynamics to draw quantitative novel insights into cell wall enzymatic hydrolysis.
• The datasets can serve as benchmarks for assessing algorithms and softwares devised to process 4D datasets, develop mathematical models, and to implement image processing softwares based on deep learning. Publicly available expert-validated 4D datasets on cell wall deconstruction remain very limited.

## 2 Background

The plant cell wall is recalcitrant to enzymatic deconstruction. The recalcitrance contributes to the high conversion costs of transformation of lignocellulosic biomass to bioproducts [1]. An in-depth understanding of enzymatic deconstruction is essential to address recalcitrance and achieve economic viability of bioproducts [2]. While recalcitrance has been widely investigated at nanoscale, it remains relatively understudied at microscale. This is mainly due to lack of microscale datasets, whose collection under enzymatic deconstruction is both time-consuming and technically demanding. In addition, analysis of such datasets is challenging, specifically due to sample drift. The relevance of microscale investigation of cell wall enzymatic hydrolysis has been recently demonstrated [3,4]. Furthermore, an efficient drift compensation method, named AIMTrack, was developed enabling accurate quantification of cell wall autofluorescence intensity dynamics and morphodynamics [5]. These dynamics were shown to be reliable indicators of saccharification dynamics [5]. The development of AIMTrack and associated research enabled collection of the time-lapse image datasets that are presented here. These datasets provide a valuable resource for extending the analysis of cell wall deconstruction. They also ensure transparency and reproducibility. They can be used as benchmarks for evaluating 4D processing algorithms, developing deep learning-based analysis tools and mathematical models.

## 3 Data Description

**Fig. 1:**
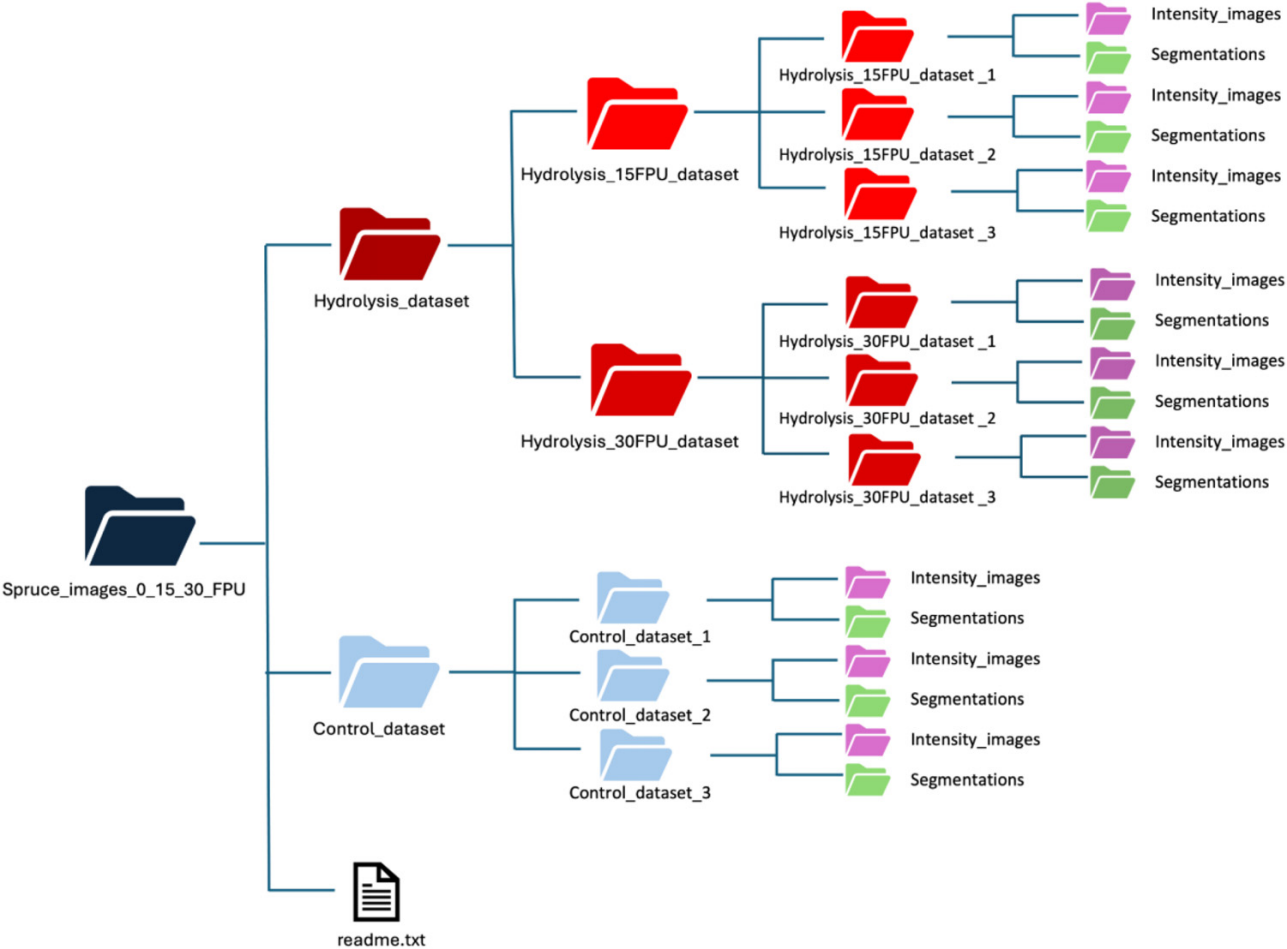
The control and hydrolysis image datasets, with enzyme loading of 15 and 30 FPU/g biomass are organized into folders and subfolders.

The control and hydrolysis 4D datasets collected with enzyme loadings of 15 and 30 FPU/g biomass together with segmentations generated with AIMTrack are organized into folder and subfolders (Fig. 1) with the following hierarchy of folders and subfolders [8]:

• Spruce_images_0_15_30_FPU
  The parent folder which includes the hydrolysis and control datasets
  ○ Hydrolysis_dataset
    Includes two hydrolysis datasets collected with 15 and 30 FPU/g biomass enzyme loadings:
    ■ Hydrolysis_15FPU_dataset
      Includes three time-lapse datasets acquired with 15FPU/g biomass:
      • Hydrolysis_15FPU_dataset_1
      • Hydrolysis_15FPU_dataset_2
      • Hydrolysis_15FPU_dataset_3
      • Hydrolysis_30FPU_dataset
        Includes three time-lapse datasets acquired with 30 FPU/g biomass:
      • Hydrolysis_30FPU_dataset_1
      • Hydrolysis_30FPU_dataset_2
    ■ Hydrolysis_30FPU_dataset_3
  ○ Control_dataset
    Includes three control datasets acquired with buffer without adding enzyme solution:
    ■ Control_dataset_1
    ■ Control_dataset_2
    ■ Control_dataset_3
  ○ readme.txt
    Includes the data organization described here together with voxel dimensions.

The terminal folders, ***Intensity_images*** and ***Segmentations,*** contain 3D acquisitions of cell wall autofluorescence intensity and corresponding segmentations obtained using AIMTrack, respectively.

## 4 Experimental Design, Materials and Methods

Experimental design involved spruce (*Picea abies*) wood sample preparation, pretreatment and time-lapse imaging using a fluorescence confocal microscope [6]. Blocks of the spruce wood, provided by INRAE Grand-Est, France, were first cut into fragments measuring 0.5 × 0.5 × 1 cm and pretreated using sodium chlorite pretreatment for partial lignin removal and improved subsequent enzymatic hydrolysis efficiency [5,7]. Specifically, the samples were immersed in eight consecutive treatment baths (1 h each) at 70°C in a solution composed of 1.25 g sodium chlorite (Sigma-Aldrich, analytical grade), 150 µL acetic acid (Carlo Erba, analytical grade), and 40 mL water [5,7]**.** The pretreatment was performed under vacuum conditions to ensure a more uniform and homogeneous impact of pretreatment on the samples. To reduce sample spatial heterogeneity which can be introduced by differential pretreatment impact, the 3 mm terminal regions along the 1 cm longitudinal axis of the pretreated samples were selected for sectioning.

Pretreated wood fragments were cut into 40 µm-thick cross sections using a microtome (HM340E, Thermo Scientific). The sections were then pre-incubated in a Gene Frame together with 60 µL of acetate buffer (pH 5, 50 mM) for 30 min at room temperature. After this pre-incubation, the buffer was removed and replaced with a cellulolytic enzyme solution at two separate activity levels of 15 and 30 FPU/g biomass. A cellulolytic enzyme cocktail (provided by IFPEN, Rueil-Malmaison, France, and produced by Trichoderma reesei) was used, including a core cellulase system (cellulase and β-glucosidase) together with hemicellulolytic and accessory activities (xylanase, mannanase, β-xylosidase, arabinosidase, and acetyl esterase). The cocktail was diluted in 50 mM acetate buffer (pH 5) to achieve final loadings of 15 and 30 FPU per g biomass. For the control image datasets acquired subsequently, the buffer was not replaced with the enzyme solution. A coverslip was then used to seal the gene frame. The glass slide was placed inside a temperature-controlled incubation chamber whose temperature was stabilized at 50 °C and mounted on the stage of a fluorescence confocal microscope (Fluoview FV3000, Olympus). Time-lapse imaging was carried out during 24 h with hourly acquisitions using a 405 nm laser at 10% power (emission range: 415–515 nm), scanned at 4 μs/pixel with 512 × 512-pixel resolution, 600 V gain, and a 50 μm confocal aperture. A 20 × objective (zoom 2 ×) was used for time-lapse imaging. Z-stacks consisted of 400 slices with a z-step set to 0.3 μm. For each enzymatic activity and control conditions, three time-lapse image datasets were acquired.

**Fig. 2:**
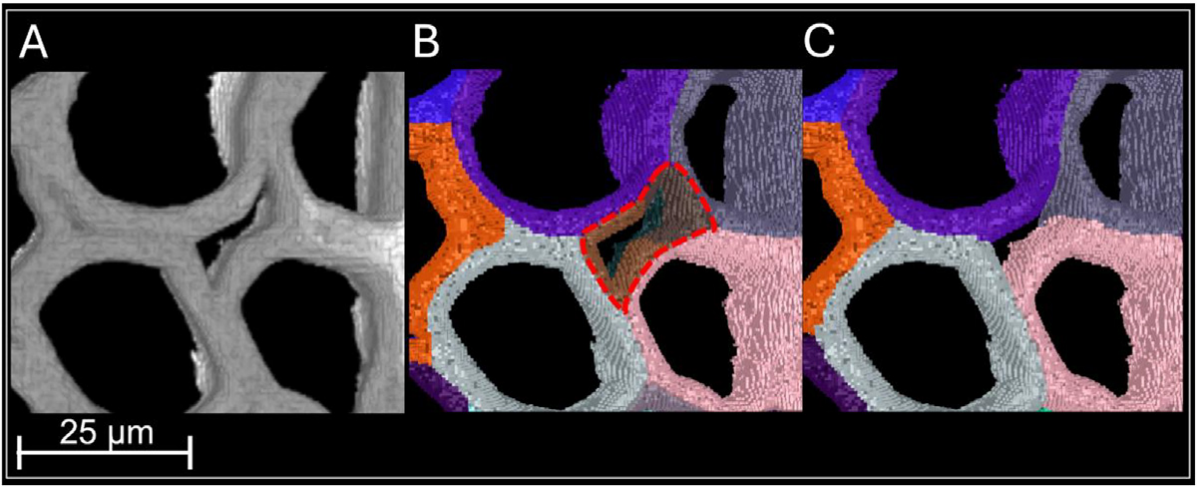
Optimal initial segmentation obtained using tuned parameters, followed by manual correction where necessary. (A) Close-up of a selected region from the initial image. (B) Segmentation result corresponding to (A), illustrating segmentation errors enclosed by dashed lines. (C) Segmentation obtained after manual correction.

Following the acquisition of the time-lapse images, AIMTrack [5] was used to process the images. AIMTrack includes an adaptive drift compensation method which divides the time-lapses into sequential clusters of consecutive images. The number of images within each cluster is adapted to the extent of deconstruction with small clusters including images exhibiting significant enzyme-induced cell wall changes and large clusters with relatively less pronounced cell wall deconstruction. The transformations are computed for intra-cluster images by pair-wise image registrations. Composition of these transformations enables sample drift and deformation compensation which is used to compute the consistently imaged region in each time-lapse image. The consistently imaged region is the spatial region of the sample which is continuously imaged in time-lapse acquisitions despite the sample drift and deformation. To segment the time-lapse images, AIMTrack first segments the initial time-lapse using a watershed algorithm and thresholding to identify the individual cell walls. Special care is taken in choosing the parameter values to achieve an optimal segmentation. Manual corrections are applied to this initial image segmentation when necessary (Fig. 2). The initial segmentation is then used to compute the segmentations of subsequent images of the time-lapse data using a propagation strategy using the previously computed transformations.

## Limitations

None.

## Ethics Statement

The authors have read and followed the ethical requirements for publication in Data in Brief and confirmed that the current work does not involve human subjects, animal experiments, or any data collected from social media platforms.

## CRediT authorship contribution statement

**Solmaz Hossein Khani:** Conceptualization, Data curation, Formal analysis, Investigation, Methodology, Software, Validation, Visualization, Writing – original draft, Writing – review & editing. **Maxime Corré:** Investigation, Methodology. **Khadidja Ould Amer:** Data curation, Methodology. **Noah Remy:** Investigation, Methodology, Writing – review & editing. **Berangère Lebas:** Investigation, Methodology. **Anouck Habrant:** Investigation, Methodology, Writing – review & editing. **Gabriel Paës:** Conceptualization, Formal analysis, Funding acquisition, Investigation, Methodology, Project administration, Resources, Supervision, Validation, Writing – original draft, Writing – review & editing. **Yassin Refahi:** Conceptualization, Data curation, Formal analysis, Funding acquisition, Investigation, Methodology, Project administration, Resources, Software, Supervision, Validation, Visualization, Writing – original draft, Writing – review & editing.

## Data Availability

Zenodo4D (Space + Time) Datasets of Spruce Wood Enzymatic Hydrolysis (Original data)

## References

[1] McCann M.C., Carpita N.C. (2015). Biomass recalcitrance: A multi-scale, multi-factor, and conversion-specific property. J. Exp. Bot..

[2] Zhang R., Gao H., Wang Y., He B., Lu J., Zhu W., Peng L., Wang Y. (2023). Challenges and perspectives of green-like lignocellulose pretreatments selectable for low-cost biofuels and high-value bioproduction. Bioresour. Technol..

[3] Refahi Y., Zoghlami A., Viné T., Terryn C., Paës G. (2024). Plant cell wall enzymatic deconstruction: bridging the gap between micro and nano scales. Bioresour. Technol..

[4] Hossein Khani S., Ould Amer K., Remy N., Lebas B., Habrant A., Faraj A., Malandain G., Paës G., Refahi Y. (2025). A distinct autofluorescence distribution pattern marks enzymatic deconstruction of plant cell wall. New Biotechnol..

[5] Hossein Khani S., Ould Amer K., Shiasi Ghalemaleki F., Corré M., Remy N., Habrant A., Lebas B., Massah J., Faraj A., Paës G., Refahi Y. (2026). Plant cell wall enzymatic hydrolysis: predicting yield dynamics from autofluorescence and morphological temporal changes. Bioresour. Technol..

[6] Zoghlami A., Refahi Y., Terryn C., Paës G. (2020). Three-dimensional imaging of plant cell wall deconstruction using fluorescence confocal microscopy. Sustain. Chem..

[7] Hossein Khani S., Remy N., Ould Amer K., Lebas B., Habrant A., Paës G., Refahi Y. (2025). Time-lapse 3D image datasets of spruce tree wood enzymatic deconstruction. Data Brief.

[8] Hossein Khani S., Corré M., Ould Amer K., Remy N., Lebas B., Habrant A., Paës G., Refahi Y. 4D (space + time) datasets of spruce wood enzymatic hydrolysis [Data set]. Zenodo.

